# Sustained hypotension during shoulder arthroscopy is associated with postoperative nausea and vomiting

**DOI:** 10.1371/journal.pone.0357794

**Published:** 2026-09-08

**Authors:** Rili Yu, Xia Yang, Juan Chen, Fan Zhang

**Affiliations:** 1 Department of Anesthesiology, The Third Xiangya Hospital, Central South University, Changsha, Hunan, People's Republic of China; 2 Department of Endocrinology, The First Hospital of Changsha, Changsha, Hunan, People's Republic of China; No institution, UNITED KINGDOM OF GREAT BRITAIN AND NORTHERN IRELAND

## Abstract

**Background:**

Postoperative nausea and vomiting (PONV) is a common and distressing complication following shoulder arthroscopy. Although hypotension is known to trigger nausea, the specific association between sustained intraoperative hypotension (SIH) during shoulder arthroscopic surgery and PONV has not been well characterized. This retrospective study aimed to investigate whether SIH is independently associated with PONV.

**Methods:**

We retrospectively analyzed data from 306 patients who underwent shoulder arthroscopy between March 2018 and January 2024. SIH was defined as a ≥ 20% decrease in mean arterial pressure (MAP) from baseline persisting for at least 60 minutes during the controlled hypotension phase. Univariate logistic regression analysis and multivariate exact logistic regression analysis were performed to identify factors associated with the occurrence of PONV. PONV severity was compared between the SIH and non-SIH groups using the chi-square test.

**Results:**

PONV occurred in 96 patients (31.37%). Univariate logistic regression analysis revealed that females, smoking, history of motion sickness and/or PONV, and SIH were related to the occurrence of PONV (all p < 0.05). Multivariate logistic regression analysis revealed that females (adjusted OR: 3.966, 95% CI: 2.149–7.317, p < 0.001), smoking (adjusted OR: 0.232, 95% CI: 0.107–0.501, p < 0.001), history of motion sickness and/or PONV (adjusted OR: 32.393, 95% CI: 3.801–276.051, p = 0.001), and SIH (adjusted OR: 2.198, 95% CI: 1.289–3.749, p = 0.004) were independently associated with the occurrence of PONV. Additionally, patients with SIH exhibited significantly higher severity grades for both nausea (p = 0.002) and vomiting (p = 0.007).

**Conclusion:**

SIH due to controlled hypotension during shoulder arthroscopic surgery was independently associated with PONV. SIH may serve as a clinically relevant predictor for PONV.

## Introduction

Postoperative nausea and vomiting (PONV) is the most common complication following surgical anesthesia, with an incidence rate of about 30% in the general population, rising to as high as 80% in high-risk groups [1]. PONV is a very distressing experience that can lead to wound dehiscence, electrolyte imbalance, and aspiration, significantly impacting patients’ satisfaction [2]. Furthermore, PONV is associated with delayed postoperative recovery, prolonged hospital stays, and increased healthcare costs [3]. Therefore, effective prevention and treatment of PONV is a critical component in the practice of enhanced recovery after surgery. The clear identification of the risk factors for PONV is a prerequisite for effective prevention and treatment. Patient-related factors include young females, non-smokers, a history of motion sickness, and/or a history of PONV [4–6]. Surgical factors encompass laparoscopic surgery, cesarean section, and otolaryngology procedures [7–9]. Anesthetic factors include volatile anesthetics, opioid analgesics, tramadol, and other opioids [10–12]. However, despite active interventions and management of the aforementioned factors, a number of patients still suffer from relatively severe PONV. This suggests that we might have overlooked other potential risks for PONV during the perioperative period.

As is well known, hypotension can induce nausea and vomiting. Hypoperfusion of the brainstem and intestine is recognized as a possible mechanism for PONV in hypotensive patients [13]. Interestingly, studies have reported an association between a reduction in the absolute value of mean arterial pressure during surgery (< 50 mmHg) and an elevated occurrence of PONV [13,14]. However, an absolute mean arterial pressure of less than 50 mmHg during surgery is an infrequent event and poses a significant risk of inadequate perfusion to vital organs, including the heart and brain. Another study confirms that a decrease in systolic blood pressure of more than 35% from baseline during the induction of anesthesia is associated with PONV, but that hypotension during the maintenance phase is not significantly related to PONV, and the reason is unclear [15]. However, intraoperative hypotension is generally defined as a decrease in blood pressure of more than 20% from baseline [16], and the duration of the hypotensive episodes should also be taken into account. The relationship between controlled, sustained intraoperative hypotension (SIH) during shoulder arthroscopy—a procedure characterized by the beach-chair position and deliberately induced and maintained hypotension—and PONV has not been specifically investigated. This study aims to investigate the association between SIH during shoulder arthroscopy and PONV, with the goal of informing more effective management strategies for PONV.

## Methods

### Study design and participants

This was a retrospective, observational, single-center cohort study. The study population consisted of patients who had exclusively undergone shoulder arthroscopic surgery at the Third Xiangya Hospital of Central South University between March 20, 2018, and January 31, 2024. The inclusion criteria were as follows: patients aged between 18 and 75 years, those who received general anesthesia, those with complete blood pressure recordings throughout the surgical procedure, those who used patient-controlled intravenous analgesia (PCIA) pumps following surgery, and those with detailed postoperative follow-up records regarding nausea and vomiting. The exclusion criteria were patients who underwent a composite surgery involving shoulder arthroscopy and those with incomplete clinical data.

This study was performed according to the guidelines of the Helsinki Declaration and approved by the Institutional Review Board of the Third Xiangya Hospital of Central South University (Date: 28/04/2024, Approval No.: K24371). The requirement for informed consent was waived for retrospectively collected data, in compliance with both national legislation and institutional guidelines. To protect the privacy of participants in this study, the personal identification data of the enrolled individuals was anonymized and replaced with a coding system.

### Data collection

The data access date for this research was set as June 26th, 2024. Intraoperative blood pressure and anesthesia-related variables were extracted by a researcher blinded to PONV outcomes, while PONV data were extracted by another researcher blinded to blood pressure data and SIH classification. Thus, exposure and outcome data were collected independently. However, owing to the retrospective design, clinicians who originally documented PONV during routine postoperative care were not blinded to the study hypothesis. In addition, a third researcher carried out data monitoring and source-data verification in strict accordance with a pre-established plan.

We collected baseline data of all selected patients including demographic data: age, gender, height, weight, smoking history, history of motion sickness and/or PONV. Intraoperatively, we considered factors such as inhalation anesthetics and opioid dosage, the duration of procedure and controlled hypotension, MAP and liquid intake. Postoperative follow-up included assessment of PONV and pain, and postoperative opioid dosage. Among them, postoperative pain was considered a potential post-outcome variable. It was considered only for univariate descriptive analysis, and was excluded from the primary multivariate regression model to prevent timing-related bias.

### Anesthesia management

Anesthesia was managed by experienced and specialized anesthesiologists. All participants received combined intravenous and sevoflurane inhalation anesthesia. They underwent shoulder arthroscopic procedures in the beach‑chair position, and received an interscalene block. The opioid analgesics used during the operation, including sufentanil, oxycodone, dezocine, and other opioids were converted to equivalent doses of sufentanil. Controlled hypotension, which involves maintaining the systolic blood pressure within a range of 90–100 mmHg, is essential for minimizing intraoperative blood loss and enhancing visualization of shoulder arthroscopy. Under the premise of maintaining appropriate sedation and analgesia, if the systolic blood pressure was still above 100 mmHg, nitroglycerin was employed to maintain the patient's systolic blood pressure within a targeted range of 90–100 mmHg through intravenous infusion. Vasoactive drugs (such as norepinephrine or ephedrine) were primarily used to correct hypotension that fell below the target or caused hemodynamic instability. All patients received 8 mg of ondansetron intravenously thirty minutes before the end of surgery to prevent PONV, without any other antiemetics during the procedure. For postoperative analgesia, all patients were equipped with PCIA pumps delivering sufentanil.

### Blood pressure assessment

Baseline blood pressure was defined as the average of three readings recorded upon patient admission. Intraoperatively, invasive arterial blood pressure monitoring was employed, with periodic recordings every five minutes. A 20% decrease in MAP from baseline is a widely accepted threshold [16]. In addition, previous literature has reported that the duration of hypotension is associated with PONV [20]. The duration of controlled hypotension during shoulder arthroscopy ranges from 60 to 80 minutes based on the retrospective data from this study. A ≥ 60-minute duration captured sustained hypotension relevant to this specific surgery. Thus, SIH was defined as a decrease in MAP of at least 20% below baseline that persisted for 60 minutes or more during the controlled hypotension phase. To assess the robustness of the results to variations in SIH definition, we conducted sensitivity analyses using different MAP reduction thresholds and duration cutoffs: (1) Model A: MAP drop ≥20% from baseline lasting for ≥30 minutes. (2) Model B: MAP drop ≥30% from baseline lasting for ≥60 minutes.

### PONV assessment

In our institution, postoperative assessments in the post-anesthesia care unit (PACU) and wards are conducted by nursing staff and anesthesiologists following strict standard operating procedures. Vital signs and subjective symptoms (including PONV) are required to be recorded at specific time points: upon admission to and discharge from the PACU, and in the ward at 24 and 48 hours after surgery. The assessment was based on the documentation provided by the attending healthcare professionals, which included the timing and severity of PONV symptoms. The postoperative nausea scale was categorized as follows: 0-absence of nausea; 1-no nausea at rest, experienced with movement; 2-infrequent nausea at rest; 3-persistent nausea at rest, with severe nausea upon movement. The postoperative vomiting scale was rated as: 0-no vomiting; 1-mild vomiting, occurring one to two times daily; 2-moderate vomiting, happening three to five times daily; 3-severe vomiting, more than six times daily. In cases where the nausea and vomiting scores were both zero, the patient was deemed to have no postoperative nausea and vomiting (n-PONV); otherwise, PONV was considered present. The administration of rescue antiemetics (such as metoclopramide or tropisetron) is rigorously documented in the electronic medical record. In this study, the administration of any rescue antiemetic was automatically categorized as a “PONV present” event, ensuring that patients who received treatment but whose detailed symptom scores might have been incompletely documented were still correctly identified.

### Postoperative pain evaluation

The patient's level of pain was evaluated using the numeric rating scale (NRS) at 24 hours and 48 hours after surgery, wherein patients were prompted to select a numerical value that most closely corresponds to their pain level, both at rest and during movement. The NRS scores are classified as follows: 0: no pain; 1–3: mild pain; 4–6: moderate pain; 7–10: severe pain.

### Statistical analysis

Statistical analyses were performed using SPSS 25.0 (SPSS Inc., Chicago, IL, USA) and SAS 9.4 (SAS Institute, Cary, NC, USA). The Kolmogorov–Smirnov test was utilized to determine whether the continuous variables conformed to a normal distribution. For continuous variables with a normal distribution, the data were presented as means±standard deviations; for non–normally distributed continuous variables, they were presented as medians and interquartile ranges; and for enumeration data, they were presented as frequencies. To assess the differences between two groups of continuous variables, the unpaired t test was employed for variables with a normal distribution, while the Mann–Whitney U test was utilized for those with a non-normal distribution. For enumeration data, the χ^2^ test was used for comparison.

Univariate logistic regression analysis was employed to screen perioperative factors for their association with PONV. Variables with a p-value <0.10 in univariate analysis were considered for inclusion in the multivariate model. Multicollinearity was assessed using Variance Inflation Factor (VIF); all VIF values in the final model were below 2.0, indicating no concerning multicollinearity among the four independent variables (female sex, smoking, history of motion sickness/PONV, and SIH). The final model included 4 independent variables with 96 PONV events, yielding an EPV ratio of 24, which substantially exceeds both the conventional minimum threshold of 10 events per variable and the more stringent criterion of 20, ensuring stable parameter estimates and reliable confidence intervals. Given the sparse data observed for the history of motion sickness/PONV variable, which caused quasi-complete separation in standard logistic regression, multivariate exact logistic regression was employed to provide more reliable estimates in the presence of small cell sizes. The area under the receiver operating characteristic curve (AUC-ROC) was used to assess model discrimination, with AUC > 0.7 indicating adequate discrimination. Sensitivity analyses were performed using alternative definitions of SIH: Model A (≥20% MAP drop for ≥30 minutes) and Model B (≥30% MAP drop for ≥60 minutes). A p-value <0.05 was considered statistically significant.

## Results

**Fig 1:**
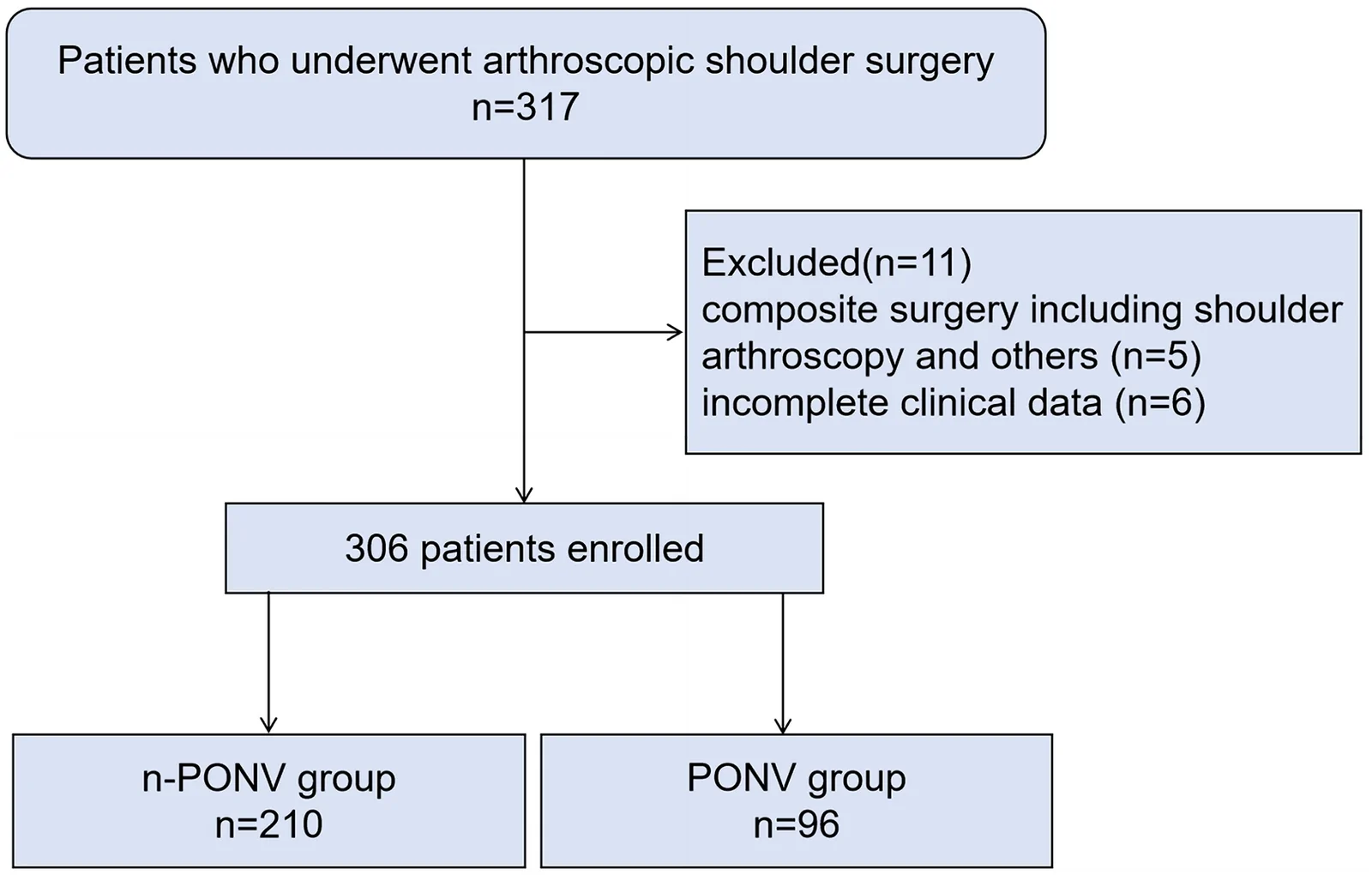
Flow chart for patient selection.

In our study, a total of 306 patients who underwent shoulder arthroscopic surgery were included. PONV was observed in 96 patients, whereas it was absent in 210 patients (Fig 1). The incidence rate of PONV was 31.37%. Although controlled hypotension was performed during the surgery, no adverse neurological sequelae, including sensory, motor, or cognitive impairments, were observed in any patient in this study.

### Baseline demographic and clinical characteristics of patients

In the present study, patients with PONV exhibited a higher proportion of females (70 [72.92%] of 96 patients in the PONV group vs. 119 [56.67%] of 210 patients in the n-PONV group), a history of motion sickness and/or PONV (8 [8.33%] of 96 patients in the PONV group vs. 1 [0.48%] of 210 patients in the n-PONV group), and SIH (61 [63.54%] of 96 patients in the PONV group vs. 91 [43.33%] of 210 patients in the n-PONV group) (all p < 0.01), but a lower proportion of smokers (11 [11.46%] of 96 patients in the PONV group vs. 49 [23.33%] of 210 patients in the n-PONV group) (p < 0.05). There were no significant differences in other demographic and clinical parameters, including age, weight, height, duration of procedure, intraoperative sevoflurane and analgesic dosages, liquid intake, duration of controlled hypotension, postoperative analgesic dosage and pain (all p > 0.05) (Table 1).

**Table 1:** Comparison of demographic characteristics and clinical data between groups.

Variables	n-PONV	PONV	Statistic	*p*-value
Patients, n	210	96		
Age (years)	57.56 ± 11.08	57.72 ± 10.02	0.122	0.903
Male / female, n	91 / 119	26 / 70	7.367	0.007^#^
Weight (kg)	61.92 ± 11.22	61.14 ± 11.32	0.563	0.574
Height (cm)	161.50 ± 8.31	160.30 ± 8.52	1.213	0.226
Smoking, n(%)	49 (23.33)	11 (11.46)	5.894	0.015*
History of motion sickness and/or PONV, n(%)	1 (0.48)	8 (8.33)	14.250	<0.001^#^
Duration of procedure (min)	143.80 ± 43.37	138.00 ± 43.36	1.091	0.276
Intraoperative sevoflurane dosage (ml)	17.14 ± 5.43	16.56 ± 5.20	0.879	0.380
Intraoperative analgesics dosage (equivalent dose of sufentanil) (μg/kg)	0.65 ± 0.05	0.64 ± 0.04	0.864	0.388
Liquid intake (ml)	1706.00 ± 377.60	1642.00 ± 458.20	1.277	0.203
Duration of controlled hypotension (min)	80.32 ± 40.38	76.93 ± 36.42	0.704	0.482
SIH, n (%)	91 (43.33)	61 (63.54)	10.760	0.001^#^
Postoperative analgesics dosage (equivalent dose of sufentanil) (μg/kg)	1.60 ± 0.16	1.60 ± 0.11	0.309	0.758
Postoperative pain			2.309	0.315
no pain, n (%)	26 (12.38)	14 (14.58)		
mild pain, n (%)	147 (70.00)	59 (61.46)		
moderate to severe pain, n (%)	37 (17.62)	23 (23.96)		

For continuous variables (age, weight, height, duration of procedure, intraoperative sevoflurane dosage, intraoperative analgesics dosage, liquid intake, duration of controlled hypotension): normally distributed data were analyzed using the unpaired t test (reported as t value). For categorical variables (gender, smoking, history of motion sickness and/or PONV, SIH, postoperative pain), the χ² test was used. Data are expressed as mean±standard deviation or number (%). PONV, postoperative nausea and vomiting; SIH, sustained intraoperative hypotension. * *p < 0.05*, ^#^ *p < 0.01*.

### The relationship between the perioperative factors and the occurrence of PONV

Univariate logistic regression analysis was utilized to explore the association between perioperative factors and the occurrence of PONV among patients undergoing shoulder arthroscopy. It showed that females (OR: 2.059, 95% CI: 1.216–3.485), smokers (OR: 0.425, 95% CI: 0.210–0.860), history of motion sickness and/or PONV (OR: 19.000, 95% CI: 2.341–154.186), and SIH (OR: 2.279, 95% CI: 1.386–3.747) were related to the occurrence of PONV (Table 2). Other factors, including patient demographics (age, weight, height), procedural details (duration of procedure, intraoperative sevoflurane and analgesic dosages, Intraoperative use of nitroglycerin and vasopressors, liquid intake, and duration of controlled hypotension), as well as postoperative variables (postoperative analgesic dosage and pain), showed no significant association with the occurrence of PONV (all p > 0.05) (Table 2).

**Table 2:** Univariate logistic regression analysis results of factors related to the occurrence of PONV.

Variables	Unadjusted OR	95% CI	*p*-value
Age (years)	1.001	0.979–1.024	0.903
Female, n	2.059	1.216–3.485	0.007^#^
Weight (kg)	0.994	0.972–1.016	0.572
Height (cm)	0.982	0.953–1.011	0.226
Smoking, n	0.425	0.210–0.860	0.017*
History of motion sickness and/or PONV, n	19.000	2.341–154.186	0.006^#^
Duration of procedure (min)	0.997	0.991–1.003	0.276
Intraoperative sevoflurane dosage (ml)	0.980	0.936–1.025	0.379
Intraoperative analgesics dosage (equivalent dose of sufentanil) (μg/kg)	0.098	0.000–19.090	0.387
Intraoperative use of nitroglycerin	1.273	0.515–3.144	0.601
Intraoperative use of vasopressors	1.286	0.605–2.734	0.514
Liquid intake (ml)	1.000	0.999–1.000	0.202
Duration of controlled hypotension (min)	0.998	0.992–1.004	0.481
SIH, n	2.279	1.386–3.747	0.001^#^
Postoperative analgesics dosage (equivalent dose of sufentanil) (μg/kg)	0.768	0.144–4.086	0.757
Postoperative pain, n	0.828	0.411–1.666	0.596

PONV, postoperative nausea and vomiting; SIH, sustained intraoperative hypotension. * *p < 0.05*, ^#^ *p < 0.01*.

Multivariate exact logistic regression analysis revealed that females (OR: 3.966, 95% CI: 2.149–7.317, *p < 0.001*), smoking (OR: 0.232, 95% CI: 0.107–0.501, *p < 0.001*), history of motion sickness and/or PONV (OR: 32.393, 95% CI: 3.801–276.051, *p* = 0.001), and SIH (OR: 2.198, 95% CI: 1.289–3.749, *p* = 0.004) were independently associated with the occurrence of PONV in patients who underwent shoulder arthroscopy (Table 3). The AUC was 0.730 (95% CI: 0.667–0.793), indicating acceptable discrimination.

**Table 3:** Multivariate logistic regression analysis results of the occurrence of PONV.

Variables	β	SE	Adjusted OR	95% CI	*p*-value
Female, n	1.378	0.312	3.966	2.149–7.317	<0.001^#^
Smoking, n	−1.461	0.393	0.232	0.107–0.501	<0.001^#^
History of motion sickness and/or PONV, n	3.478	1.093	32.393	3.801–276.051	0.001^#^
SIH, n	0.788	0.272	2.198	1.289–3.749	0.004^#^

PONV, postoperative nausea and vomiting; SIH, sustained intraoperative hypotension. * *p < 0.05*, ^#^ *p < 0.01*. Multivariate model was re-analyzed using exact logistic regression. All VIF values were below 2.0, indicating no concerning multicollinearity. The EPV ratio was 24:1.

Sensitivity analysis indicated that model A (MAP drop ≥20% from baseline lasting for ≥30 minutes) showed a weak association with the occurrence of PONV (OR: 1.710, 95% CI: 1.041–2.808, p = 0.034). Model B was similar to model A (OR: 1.763, 95% CI: 1.080–2.878, p = 0.023), but model B had limited statistical power due to a smaller sample size meeting the stricter threshold.

### SIH and severity of PONV

There was a statistically significant difference in nausea severity between the two groups (χ² = 14.510, p = 0.002). In the n-SIH group, 119 patients reported absence of nausea (Grade 0), while 19 patients experienced nausea only with movement (Grade 1), 14 patients had infrequent nausea at rest (Grade 2), and only 2 patients had persistent nausea with severe nausea upon movement (Grade 3). In contrast, the SIH group showed a higher proportion of patients with more severe nausea: 91 patients had no nausea, 22 had Grade 1 nausea, 29 had Grade 2 nausea, and 10 had Grade 3 nausea (Table 4).

**Table 4:** Comparison of PONV severity between the SIH and n-SIH groups.

Variables	n-SIH	SIH	χ^2^	*p*-value
Postoperative nausea grading, n			14.510	0.002^#^
0-absence of nausea	119	91		
1-no nausea at rest, experienced with movement	19	22		
2-infrequent nausea at rest	14	29		
3-persistent nausea at rest, with severe nausea upon movement	2	10		
Postoperative vomiting grading, n			12.070	0.007^#^
0-no vomiting	122	98		
1-mild vomiting, occurring one to two times daily	18	21		
2-moderate vomiting, happening three to five times daily	13	25		
3-severe vomiting, more than six times daily	1	8		

PONV, postoperative nausea and vomiting; SIH, sustained intraoperative hypotension. * *p < 0.05*, ^#^ *p < 0.01*.

For postoperative vomiting grading: A statistically significant difference was also observed in vomiting severity between the two groups (χ² = 12.070, p = 0.007). In the n-SIH group, 122 patients reported no vomiting (Grade 0), 18 had mild vomiting (1–2 times daily, Grade 1), 13 had moderate vomiting (3–5 times daily, Grade 2), and only 1 patient had severe vomiting (>6 times daily, Grade 3). In the SIH group, 98 patients had no vomiting, 21 had Grade 1 vomiting, 25 had Grade 2 vomiting, and 8 patients had severe vomiting (Grade 3) (Table 4).

Furthermore, we performed a stratified analysis to differentiate early PONV (occurring in the PACU, 0–2 hours after surgery) from late PONV (occurring within 2–48 hours postoperatively). Our analysis showed that SIH was significantly associated with early PONV (OR: 2.079, 95% CI: 1.252–3.454, *p* = 0.005) and late PONV (OR: 2.448, 95% CI: 1.467–4.086, *p* = 0.001).

## Discussion

In this study, we discovered that females, smoking, history of motion sickness and/or PONV, and SIH were independently associated with the occurrence of PONV in patients who underwent shoulder arthroscopy. Moreover, SIH showed a higher proportion of patients with more severe PONV.

PONV is one of the most common complications following surgical anesthesia, and its prevention and treatment have received high attention from anesthesiologists and surgeons. Medications used for the prevention and treatment of PONV include 5-HT3 receptor antagonists, dexamethasone, antihistamines, dopamine receptor antagonists, and NK1 receptor antagonists [1,17,18]. However, the prevention and treatment effects of PONV are not ideal. The occurrence of PONV is often predicted using the Apfel scores [19], which include risk factors such as females, history of motion sickness and/or PONV, and non-smoking, which is consistent with our findings. However, SIH is not included in the traditional Apfel score. The relationship between SIH and PONV is not entirely clear.

Previous limited studies have found that hypotension during general anesthesia is associated with PONV [14,15,20], but there is controversy [21], and the definition of intraoperative hypotension varies. Pusch et al. confirm that a decrease in systolic blood pressure of more than 35% from baseline during the induction of anesthesia is associated with PONV, but that hypotension during the maintenance phase is not significantly related to PONV [15]. Nakatani et al. discover that the cumulative duration of hypotension during thyroidectomy is associated with PONV [20]. Maleczek et al. through retrospective analysis, identify that intraoperative MAP below 50 mmHg for more than 1.8 minutes is a predictor for PONV [14]. Unlike these studies, our research uniquely revealed that MAP dropping below 20% of the baseline value and lasting for at least 60 minutes during shoulder arthroscopy was associated with an increased occurrence of PONV. To minimize intraoperative bleeding and enhance the visibility of the surgical field, patients are required to undergo controlled hypotension while in the beach-chair position during shoulder arthroscopy. In this position, due to the effect of gravity, cerebral perfusion pressure is significantly lower than the systemic pressure measured at arm level. This treatment can also significantly decrease local cerebral oxygen saturation in patients [22]. The above factors can directly cause relative ischemia in brainstem-related regions [14,15], reduce intestinal perfusion [22], and lead to excessive excitement of the vagus nerve and subsequent symptoms of nausea and vomiting. Our study is the first time to specifically examine PONV in the context of sustained hypotension during shoulder arthroscopy, and link both the threshold and duration of hypotension to PONV in this surgical setting. The novelty lies in the specific clinical scenario (controlled hypotension + beach-chair position), not in the general concept of hypotension causing PONV.

Our findings have several practical implications for perioperative care. Anesthesiologists should be aware that sustained hypotension during shoulder arthroscopy is not merely a hemodynamic consideration but may also be associated with clinically meaningful consequences for PONV. In particular, prolonged reductions in MAP of ≥20% from baseline lasting 60 minutes or more warrant careful attention. However, our findings do not suggest that controlled hypotension should be abandoned during shoulder arthroscopy, as it remains an important technique for reducing intraoperative bleeding and improving surgical visualization. Rather, unnecessary prolongation or excessive depth of hypotension should be avoided whenever possible. In patients with established PONV risk factors, particularly those identified by the Apfel score, closer hemodynamic monitoring and more aggressive multimodal PONV prophylaxis may be warranted. Any modification of hypotension strategies must be balanced against surgical field quality, bleeding control, cerebral perfusion, and overall patient safety. Future prospective studies are needed to determine whether optimizing hypotension management, or reducing the need for sustained controlled hypotension through strategies such as epinephrine-containing irrigation fluid, can decrease PONV without compromising surgical outcomes.

There are the following confounding factors in this study. All patients routinely received 8 mg of ondansetron intravenously thirty minutes before the end of surgery. This standardized prophylaxis may have masked the true pathophysiological effect of SIH on PONV, potentially leading to an underestimation of the association. All patients used opioid-based PCIA pumps postoperatively. Opioids are a well-known cause of PONV, and this uniform exposure may have acted as a confounder. However, the total postoperative opioid dosage did not differ significantly between the PONV and n-PONV groups (p = 0.758), suggesting that opioid use did not drive the observed association in our cohort. While we adjusted for several anesthesia-related variables (e.g., sevoflurane dosage, intraoperative opioid dosage, use of nitroglycerin and vasopressors), residual confounding from unmeasured factors (e.g., depth of anesthesia, or use of other adjunct medications) cannot be excluded.

It should be pointed out that this study has certain limitations. First, due to the retrospective nature of this study, we cannot establish causality between SIH and PONV. Our findings demonstrate an association only, and prospective randomized controlled trials are needed to confirm these results. Second, this study was conducted at a single tertiary medical center, which may limit the generalizability of our findings to other institutions or patient populations with different perioperative management protocols. Third, selection bias may be present, as only patients with complete blood pressure recordings and detailed postoperative follow-up records were included. Patients with incomplete records were excluded, and no data imputation was performed. Therefore, the potential influence of missing data on the study findings cannot be completely ruled out. In addition, outcome assessment was based on retrospective medical record review, which may have introduced information bias. Fourth, the small number of patients with a history of motion sickness and/or PONV (n = 9) led to wide confidence intervals for this predictor in the multivariate analysis, indicating model instability. Although multivariate exact logistic regression was used to address this issue, the results for this variable should be interpreted with caution. Fifth, all patients uniformly received 8 mg of ondansetron as prophylaxis. This universal intervention may have suppressed the true incidence and severity of PONV, potentially underestimating the association between SIH and PONV. Finally, neurological outcomes were not prospectively assessed; therefore, the absence of documented neurological sequelae in our retrospective data does not preclude the possibility of subclinical events.

## Conclusion

SIH due to controlled hypotension during shoulder arthroscopic surgery was independently associated with PONV.

## References

[1] Gan TJ, Belani KG, Bergese S, Chung F, Diemunsch P, Habib AS, et al. Fourth Consensus Guidelines for the Management of Postoperative Nausea and Vomiting. Anesth Analg. 2020;131(2):411–48. doi: 10.1213/ANE.0000000000004833 32467512

[2] Khanna SS, Mohammed Abdul MS, Fatima U, Garlapati H, Qayyum MA, Gulia SK. Role of general anesthetic agents in postoperative nausea and vomiting: A review of literature. Natl J Maxillofac Surg. 2022;13(2):190–4. doi: 10.4103/njms.NJMS_146_20 36051800 PMC9426712

[3] Meyer TA, Habib AS, Wagner D, Gan TJ. Neurokinin-1 receptor antagonists for the prevention of postoperative nausea and vomiting. Pharmacotherapy. 2023;43(9):922–34. doi: 10.1002/phar.2814 37166582

[4] Darvall J, Handscombe M, Maat B, So K, Suganthirakumar A, Leslie K. Interpretation of the four risk factors for postoperative nausea and vomiting in the Apfel simplified risk score: an analysis of published studies. Can J Anaesth. 2021;68(7):1057–63. doi: 10.1007/s12630-021-01974-8 33721198

[5] Rajan N, Joshi GP. Management of postoperative nausea and vomiting in adults: current controversies. Curr Opin Anaesthesiol. 2021;34(6):695–702. doi: 10.1097/ACO.0000000000001063 34560688

[6] Echeverria-Villalobos M, Fiorda-Diaz J, Uribe A, Bergese SD. Postoperative Nausea and Vomiting in Female Patients Undergoing Breast and Gynecological Surgery: A Narrative Review of Risk Factors and Prophylaxis. Front Med (Lausanne). 2022;9:909982. doi: 10.3389/fmed.2022.909982 35847822 PMC9283686

[7] Wolk RL, Lalwani AK, Pagano PP. Is dexamethasone effective in preventing nausea and vomiting after common otolaryngology procedures? Laryngoscope. 2017;127(7):1493–5. doi: 10.1002/lary.26543 28271536

[8] Tan HS, Habib AS. The optimum management of nausea and vomiting during and after cesarean delivery. Best Pract Res Clin Anaesthesiol. 2020;34(4):735–47. doi: 10.1016/j.bpa.2020.04.012 33288123

[9] Salamah HM, Elsayed E, Brakat AM, Abualkhair KA, Hussein MA, Saber SM, et al. The effects of acupressure on postoperative nausea and vomiting among patients undergoing laparoscopic surgery: A meta-analysis of randomized controlled trials. Explore (NY). 2023;19(3):300–9. doi: 10.1016/j.explore.2022.10.015 36319586

[10] Sriganesh K, Bharadwaj S, Shanthanna H, Rao GSU, Kramer BW, Sathyaprabha TN. Opioid versus non-opioid analgesia for spine surgery: a systematic review and meta-analysis of randomized controlled trials. Eur Spine J. 2023;32(1):289–300. doi: 10.1007/s00586-022-07469-4 36437435

[11] Schaefer MS, Kranke P, Weibel S, Kreysing R, Kienbaum P. Total intravenous anaesthesia versus single-drug pharmacological antiemetic prophylaxis in adults: A systematic review and meta-analysis. Eur J Anaesthesiol. 2016;33(10):750–60. doi: 10.1097/EJA.0000000000000520 27454663

[12] Derry S, Cooper TE, Phillips T. Single fixed-dose oral dexketoprofen plus tramadol for acute postoperative pain in adults. Cochrane Database Syst Rev. 2016;9(9):CD012232. doi: 10.1002/14651858.CD012232.pub2 27654994 PMC6457609

[13] Goss S, Jedlicka J, Strinitz E, Niedermayer S, Chappell D, Hofmann-Kiefer K, et al. Association between intraoperative hypotension and postoperative nausea and vomiting: a retrospective cohort study. Curr Med Res Opin. 2024;40(8):1439–48. doi: 10.1080/03007995.2024.2373885 38946490

[14] Maleczek M, Laxar D, Geroldinger A, Kimberger O. Intraoperative Hypotension Is Associated with Postoperative Nausea and Vomiting in the PACU: A Retrospective Database Analysis. J Clin Med. 2023;12(5):2009. doi: 10.3390/jcm12052009 36902796 PMC10004657

[15] Pusch F, Berger A, Wildling E, Tiefenthaler W, Krafft P. The effects of systolic arterial blood pressure variations on postoperative nausea and vomiting. Anesth Analg. 2002;94(6):1652–5, table of contents. doi: 10.1097/00000539-200206000-00054 12032046

[16] Gregory A, Stapelfeldt WH, Khanna AK, Smischney NJ, Boero IJ, Chen Q, et al. Intraoperative Hypotension Is Associated With Adverse Clinical Outcomes After Noncardiac Surgery. Anesth Analg. 2021;132(6):1654–65. doi: 10.1213/ANE.0000000000005250 33177322 PMC8115733

[17] Apfel CC, Läärä E, Koivuranta M, Greim CA, Roewer N. A simplified risk score for predicting postoperative nausea and vomiting: conclusions from cross-validations between two centers. Anesthesiology. 1999;91(3):693–700. doi: 10.1097/00000542-199909000-00022 10485781

[18] Williams BA, Holder-Murray JM, Nettrour JF, Ibinson JW, DeRenzo JS, Dalessandro C, et al. Aim for zero: prevention of postoperative nausea and vomiting using an off-patent five-drug multimodal approach. Br J Anaesth. 2023;131(1):e1–4. doi: 10.1016/j.bja.2023.01.005 36737386

[19] Sakanoue H, Yamaji H, Okamoto S, Okano K, Fujita Y, Higashiya S, et al. Incidence of nausea/vomiting following propofol sedation with adaptive servo-ventilation for atrial fibrillation ablation. J Arrhythm. 2024;40(2):289–96. doi: 10.1002/joa3.13012 38586848 PMC10995605

[20] Nakatani H, Naito Y, Ida M, Sato M, Okamoto N, Nishiwada T, et al. Association between intraoperative hypotension and postoperative nausea and vomiting: a retrospective analysis of 247 thyroidectomy cases. Braz J Anesthesiol. 2023;73(5):635–40. doi: 10.1016/j.bjane.2021.02.029 33766682 PMC10533957

[21] Kranke P, Roewer N, Rüsch D, Piper SN. Arterial hypotension during induction of anesthesia may not be a risk factor for postoperative nausea and vomiting. Anesth Analg. 2003;96(1):302–3; author reply 303. doi: 10.1097/00000539-200301000-00064 12505973

[22] Gan TJ, Mythen MG, Glass PS. Intraoperative gut hypoperfusion may be a risk factor for postoperative nausea and vomiting. Br J Anaesth. 1997;78(4):476. doi: 10.1093/bja/78.4.476 9135331

